# Absence of EEG correlates of self-referential processing depth in ALS

**DOI:** 10.1371/journal.pone.0180136

**Published:** 2017-06-29

**Authors:** Tatiana Fomina, Sebastian Weichwald, Matthis Synofzik, Jenifer Just, Ludger Schöls, Bernhard Schölkopf, Moritz Grosse-Wentrup

**Affiliations:** 1 Department of Empirical Inference, Max Planck Institute for Intelligent Systems, Tübingen, Germany; 2 International Max Planck Research School for Cognitive and Systems Neuroscience, University of Tübingen, Tübingen, Germany; 3 Department of Neurology, University of Tübingen, Tübingen, Germany; 4 German Center of Neurodegenerative Diseases (DZNE), Tübingen, Germany; 5 Hertie Institute for Clinical Brain Research, Tübingen, Germany; Centre of Genomic & Post Genomics, ITALY

## Abstract

Self-referential processing is a key cognitive process, associated with the serotonergic system and the default mode network (DMN). Decreased levels of serotonin and reduced activations of the DMN observed in amyotrophic lateral sclerosis (ALS) suggest that self-referential processing might be altered in patients with ALS. Here, we investigate the effects of ALS on the electroencephalography correlates of self-referential thinking. We find that electroencephalography (EEG) correlates of self-referential thinking are present in healthy individuals, but not in those with ALS. In particular, thinking about themselves or others significantly modulates the bandpower in the medial prefrontal cortex in healthy individuals, but not in ALS patients. This finding supports the view of ALS as a complex multisystem disorder which, as shown here, includes dysfunctional processing of the medial prefrontal cortex. It points towards possible alterations of self-consciousness in ALS patients, which might have important consequences for patients’ self-conceptions, personal relations, and decision-making.

## Introduction

Amyotrophic lateral sclerosis (ALS) is a neurodegenerative disease that is characterised mainly by the loss of motor neurons [1]. Although ALS has long been believed to be purely a motor disease, there is a growing body of physiological evidence to suggest that neuronal degeneration in ALS is not limited to motor cortices and motor pathways [2]. In particular, Braak et al. related ALS to the buildup of pTDP-43 protein agglomerations [3]; they showed that these agglomerations spread from the motor cortices to nearby areas and, eventually, to most of the cortex (with particular preponderance of the frontotemporal cortices). The broad multisite cortical damage of ALS was subsequently confirmed with a neuroimaging study by Schmidt et al. [4], demonstrating alterations in functional and structural connectivity throughout the cortex.

Given such widespread physiological alterations in the brain, it is not surprising that ALS is often accompanied by deficits in emotional and cognitive processing [5]. Cases of impaired emotions [6] have been reported in ALS patients. Zimmerman et al. found facial emotion recognition deficits in bulbar ALS [7]. Massman et al. examined 146 patients with a battery of neuropsychological tests [8] and found that ALS patients performed worse than healthy individuals in word generation, immediate free recall, attention and mental control tasks. They also found a correlation between the severity of ALS symptoms and cognitive impairment. Later studies found approximately half of the ALS patients that were examined to be cognitively impaired with alterations in particular of frontotemporal functions like memory, executive functions, judgment and reasoning [9–11].

Several studies have related the impaired cognitive functions to anatomical alterations in the prefrontal areas of the brain [9, 12–15]. Ludolph et al. found that decreased verbal fluency in ALS correlated with reduced glucose metabolism in the prefrontal cortex [12]. Abrahams et al. later confirmed the connection between decreased verbal fluency and reduced activity in the prefrontal cortex by using positron emission tomography (PET) [13] and found white matter changes in the frontal areas of the brains of ALS patients [14]. Mantovan et al. related abnormal memory retrieval to frontal lobe dysfunction by using single photon emission computer tomography (SPECT) [15].

**Fig 1:**
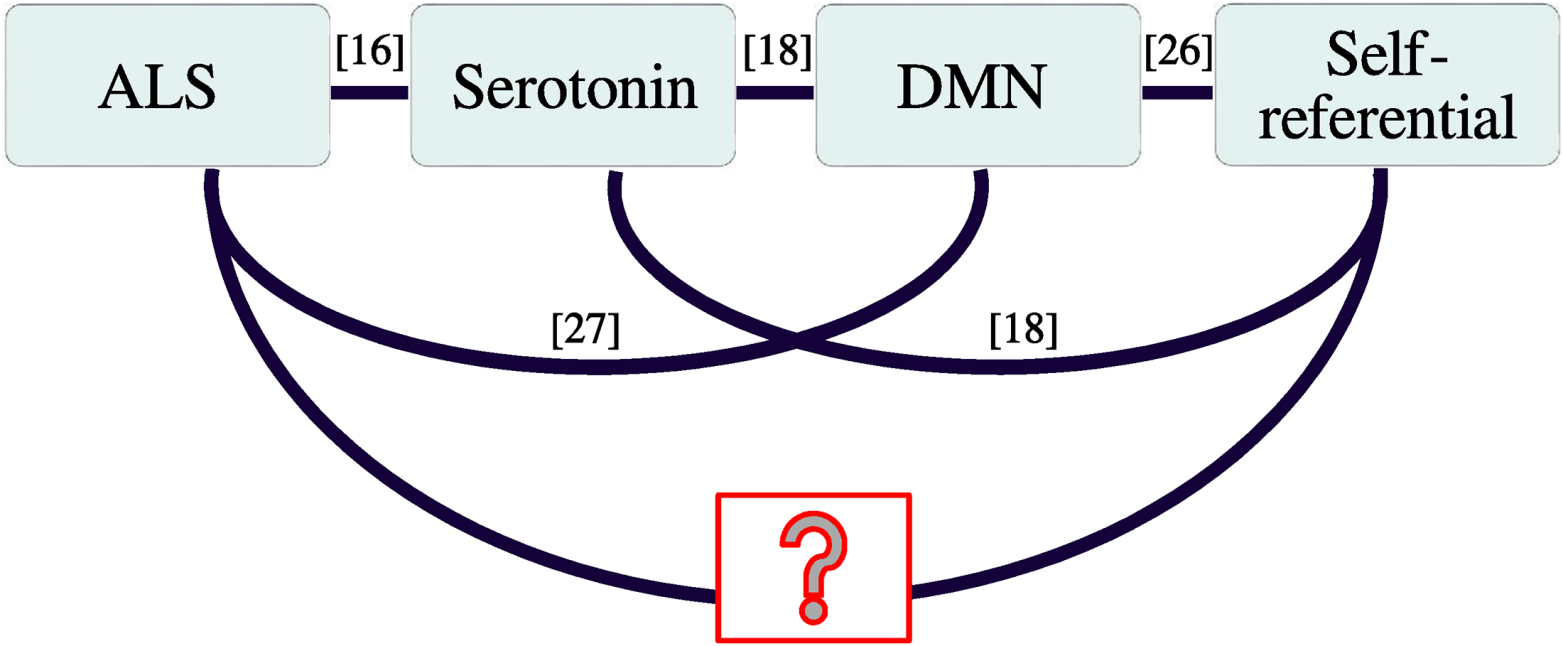
Motivation. Overview of the studies relating ALS and self-referential processing.

In addition to executive functions and memory retrieval, the prefrontal cortex is also involved in one of the main cognitive processes, namely self-referential thinking. Self-referential thinking, one of the key elements of self-awareness and consciousness, has not been investigated in ALS patients to date. Nevertheless, alterations in the prefrontal cortex (PFC) and the medial prefrontal cortex (MPFC) in particular [13, 14] lead us to hypothesise that self-referential processing may be affected in the progress of ALS. This hypothesis is further supported by ALS patients having decreased serotonin concentrations [16, 17], a neurotransmitter connected to self-referential processing [18] (Fig 1). In the following paragraphs, our motivation for this study is explained in further detail.

Recent research suggests that ALS affects deep brain structures, including the serotonergic system (Fig 1). Dentel et al. found pathological agglomerates of pTDP-43 protein in the central serotonergic neurons of the brainstem (raphe nuclei) [16]. These areas are involved in the regulation of sleep-arousal [19, 20], and their degeneration probably gives rise to the sleep disorders that are observed in ALS patients [21–23]. Moreover, the raphe nuclei release serotonin to the whole brain, and therefore one can expect the degeneration of raphe nuclei to correlate with decreased serotonin concentrations in the brain, which has indeed been observed in ALS [16, 17]. Based on the serotonin’s strong relation with locomotion (for a detailed review see [24]), Sandyk suggested a serotonergic model of ALS progression [24]. This model explains the ALS symptoms with degeneration of the serotonin projections in the motor cortices [16] and serotonin deficiency.

Serotonin-innervated neurons outside of the motor cortices are involved in high cognitive processing and, in particular, self-referential thinking (Fig 1): Hahn et al. have shown that the intensity of self-referential thinking correlates with the concentrations of serotonin receptors in the default mode network (DMN) [18]. The DMN, which comprises the precuneus/posterior cingulate cortex, MPFC and the temporoparietal junction, is a resting-state network that is active in the absence of any cognitively demanding tasks [25], and it is involved in self-referential processing [26]. Reduced serotonin projections in the DMN nodes and altered DMN activity in ALS [27] lead to the question of whether self-referential thinking is altered in ALS patients (Fig 1).

Self-referential thinking is a cognitive process and has been widely studied in healthy individuals using different experimental techniques. Kelley et al. studied blood oxygen level-dependent (BOLD) brain activation, using functional magnetic resonance imaging (fMRI) while the subjects performed trait judgments about themselves and others; the study found that the subjects selectively engaged the MPFC for self-referential judgments [28]. In a following BOLD fMRI study, Heatherton et al. found that the left MPFC also differentiates thinking about oneself and close friends [29]. D’Argembeau et al. found the cerebral metabolism in the ventral MPFC (VMPFC) to correlate with the level of self-referential processing, comparing PET measurements acquired while the subjects were thinking about themselves, others, society or relaxing [30]. Later, Whitfield-Gabrieli et al. performed a similar BOLD fMRI study comparing self-referential activations to the DMN. They suggested that the VMPFC is related to self-referential thinking in the absence of attention to external stimuli and that the dorsal MPFC (DMPFC) is related to the consideration of psychological traits in people [31].

Several electroencephalography (EEG) studies have also targeted self-referential processes (for a detailed review see [32]). Esslen et al. compared EEG recordings during judgments about self and others and found, using time-series analysis combined with source-localisation low-resolution brain electromagnetic tomography (LORETA) methods, that the VMPFC is involved in self-referential thinking in pre-self-reflective time periods while the DMPFC is involved in reflective time periods [33]. Mu et al. conducted a similar study and found that event-related desynchronisation (ERD) is related to self-referential thinking in the centro-parietal *β* (20–27 Hz), the fronto-central *γ* (28–40 Hz) and the right parieto-occipital *θ* (5–7 Hz) [34].

In the present study, we investigate self-referential processing in ALS patients. So that patients at various disease stages, ranging from early symptoms to completely locked-in state (CLIS) could be included in the analysis, we decided to use neuroimaging methods (for a review of methods see [35]). In particular, neuroimaging methods were preferred over behavioural methods, since the behavioural methods require two-way communication. For example, a memory test that was used previously by Harvey et al. to study self-referential thinking in schizophrenic patients [36] requires the patients to answer questions and cannot be used with CLIS patients who cannot communicate in any way. In order to avoid unnecessary risks associated with transporting artificially ventilated patients, we decided to use EEG, which allowed us to perform the recordings in the homes of the participants. We speculate that EEG correlates of self-referential processing may be able to detect changes in the brain at sub-clinical stages of the disease, before they become apparent from the exhibited behaviour. Tsermentseli et al. and Portet et al. previously reported similar observations whereby they found changes in the BOLD fMRI signal and ERPs both in the cognitively impaired patients and in those who showed no signs of cognitive impairment [5, 9]. Tsermentseli et al. suggested that neuroimaging alterations precede clinical symptoms in the cognitive domain. Similarly, anatomical alterations precede the decline of motor functions; muscle atrophy develops only when at least one third of the motor neurons are affected [37].

To investigate self-referential thinking with EEG, we employed a widely used setup, which allows to induce different depths of self-referential processing, ranging from thinking of oneself to a close person to a celebrity [28, 30]. During the experiment, we asked the participants to make judgments about themselves and others. We added a control non-self-referential condition, for which we asked participants to count syllables, to validate that the participants are able to follow the instructions. We additionally validated our method with healthy individuals to make sure that we are able to detect the EEG correlates of self-referential processing. We found a significant difference between log-bandpower EEG during self-referential and non-self-referential thinking both for healthy individuals and ALS patients. We further found a significant difference in log-bandpower EEG for different depths of self-referential processing in healthy individuals. Crucially, however, this effect was absent in ALS patients. This observation raises important questions regarding ALS patients’ self-conception, i.e., their ability to distinguish between themselves and others.

## Materials and methods

### Participants

EEG data were recorded from ten ALS patients (mean age 51.5 ± 11.7 years, ALSFRS-R scores [38]: 0 (CLIS), 0 (CLIS), 0 (LIS), 1, 12, 14, 17, 32, 35, 40 on a scale from 0 to 48) and ten healthy individuals (mean age 61.4 ± 6.4 years). All ALS and healthy participants were recruited from the local community (from the motor neuron disease outpatient clinic of the Department of Neurology, University of Tübingen, Germany or through Deutsche Gesellschaft für Muskelkranke e.V.), were native German speakers and were not diagnosed with any additional neurological diseases (apart from ALS).

All recordings were carried out in the participants’ homes. For safety reasons, it was recommended that severely paralysed and artificially ventilated ALS patients were not transported. Healthy individuals were visited at their homes in order to make the conditions for ALS patients and healthy individuals comparable. For severely paralysed ALS patients, all the recordings were performed in the constant presence of a caretaker.

All participants or their legal representatives gave written informed consent according to the Declaration of Helsinki and the guidelines set by the Max Planck Society and they received financial compensation for their participation. The study was approved by the Max Planck Society’s Ethics Committee.

### Hardware

EEG data were obtained using an EEG cap with 121 actiCAP active electrodes at a sampling frequency of 500 Hz and a QuickAmp amplifier (BrainProducts GmbH, Germany). The electrodes were placed according to the 10–5 system, using the electrode located over the left mastoid (TPP9h in 10–5 system) as the initial reference. All recordings were converted to a common average reference.

### Experimental design

The study design was based on similar fMRI [28, 29, 39] and EEG [34] studies with healthy individuals. We presented the participants with adjectives, as stimuli, and asked them to make judgments about whether these adjectives described the participants themselves, a friend or a celebrity; this resulted in three levels of self-referential processing depth. In order to identify the effects that were specific to self-referential processing and were not related to general cognitive decline or decreased attention, we introduced a fourth control condition that did not involve any self-referential thinking. In that condition, the participants were asked to count the syllables of the adjective that they were presented with.

Prior to the experiment, the participants were asked to choose a close friend (referred as “Friend”) and a celebrity (referred as “Celebrity”). Throughout the experiment, all stimuli were presented to the subjects aurally, through the CereProc text-to-speech system (CereProc Limited, United Kingdom). All stimuli were in German, and all participants, independent of their disease stage, received the same instructions.

In the beginning of the experiment, two consecutive periods of resting state, each of five-minute duration (eyes-open and eyes-closed), were recorded. The participants were instructed to relax and let their mind wander. In the eyes-open condition, the participants who were able to control the eye movements were additionally asked to fixate their eyes on a cross in the middle of a computer screen that was placed at a distance of 1.25 ± 0.2 m. The two resting-state datasets were used to determine the individual frequency bands, as described in [40].

The next part of the study consisted of 80 trials recorded in a single run. Each trial started with the word “Pause” (German, “Pause”) being played. During this three second-long pause, the participants were instructed to relax. After the pause, the participants heard a cue: “Selbst” (German, “Self”), “Freund” (German, “Friend”), “Prominente” (German, “Celebrity”) or “Zählen” (German, “Count”). Depending on the cue, the participants were asked either to make judgments about themselves, their friend or the celebrity, or to count the syllables of the adjective (Table 1). The adjective was then played and the participants were asked to make the appropriate judgement according to the cue that they had previously been given. All the adjectives were pseudo-randomly drawn from a list of 100 German adjectives [41].

**Table 1:** The experimental setup. Cues and correspondent activities.

Cue	Activity
Self	make judgement whether the following adjective characterises the participant himself/herself
Friend	make judgement whether the following adjective characterises the friend that the participant has selected
Celebrity	make judgement whether the following adjective characterises the celebrity that the participant has selected
Count	count syllabuses of the following adjective

Each trial had a ten-second duration (3s pause + 2s cue + 5s adjective). The participants who were able to control the eye movements were asked to fixate their eyes on the cross and to move as little as possible for the duration of the experiment.

### Data analysis

#### Individual frequency bands

The *θ* and *α* boundaries were determined individually for each subject in both the eyes-open and eyes-closed resting conditions [40]. We employed the established observation that there is more power in the *α* frequency band in the eyes-closed state than in the eyes-open state [40]. We computed the log-bandpower (fast Fourier transform (FFT) with a Hann window of five-minute width) of the channel Oz, overlapped the two log-bandpower spectra and determined the intersections around the *α* peak. The upper *θ* (lower *α*) boundary was set to the integer nearest to the first intersection point before the *α* peak. The lower *θ* was set to the half of the upper *θ* (rounded to the nearest integer). The upper *α* (lower *β*) boundary was set, to the nearest integer, to the first intersection point after the *α* peak. The upper *β* boundary was set to 30 Hz, the lower *γ* to 30–45 Hz and the upper *γ* to 55–85 Hz.

#### ICA artefact attenuation

EEG recordings are often contaminated by muscle (electromyography (EMG)) [42] and ocular (electrooculography (EOG)) artefacts [43]. We attenuated the effects of these artefacts by using second-order blind identification (SOBI) independent component analysis (ICA) [44]. Specifically, the data from each subject were first high-pass filtered with a third-order Butterworth filter with cutoff frequency of 0.1 Hz and separated into independent components (ICs). The ICs were then inspected visually and deemed to be cortical if they fulfilled the following criteria [45]: (i) the IC spectrum followed the cortical 1/f-behaviour, (ii) the IC topography was dipolar, (iii) the IC time series contained no EOG-like activity (eyelid blinks, eye movements), and (iv) the IC time series contained no other artefacts (50 Hz line noise, large spikes). Only the cortical ICs that satisfied all of these conditions were re-projected on the 121 electrodes in order to obtain clean data with the muscular, ocular and other artefacts attenuated. We obtained on average 18, 18 ± 3, 12 cortical ICs for healthy individuals and 13, 6 ± 2, 12 cortical ICs for ALS patients. The implications of different number of cortical ICs are discussed in the Discussion section.

#### Beamforming

**Fig 2:**
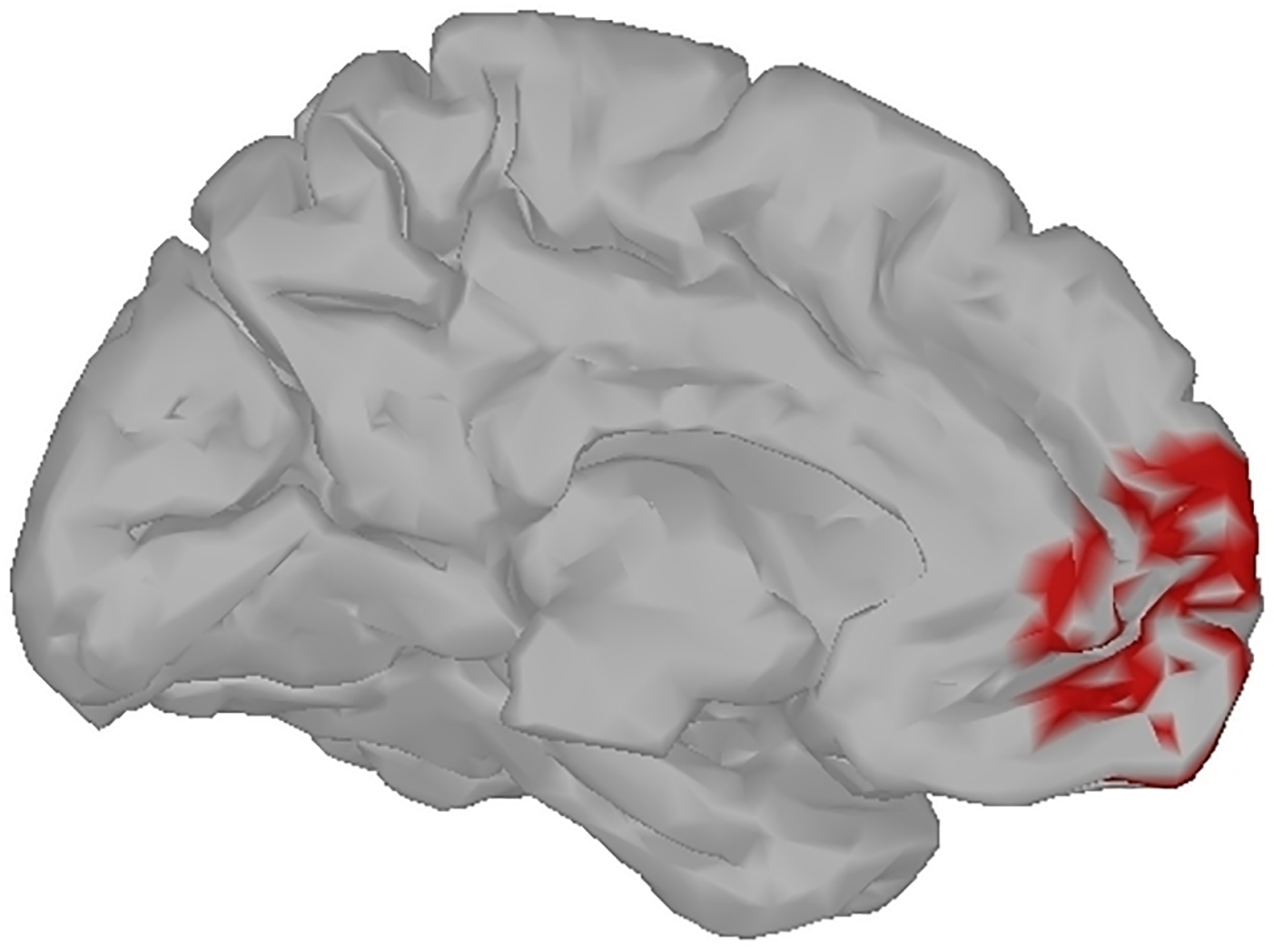
The beamformer target. Left hemisphere medial view: the voxels chosen for the beamformer are shown in red.

Following the results of a previous fMRI study of healthy individuals, in the present study we expect to see modulation in left MPFC [29]. The MPFC is situated on the inside between the two cerebral hemispheres, and thus is not directly accessible by EEG measurements taken on the surface of the scalp. The MPFC activity can be evaluated with a source localisation procedure. For this purpose, we first generated a forward model for K = 15028 dipoles spread over the cortex with the BrainStorm toolbox [46] for standardised electrode locations and a standardised three-shell spherical head model. There is no established method for localising the MPFC with EEG. Therefore, we manually selected the voxels that overlapped with areas that were found to be modulated with self-referential thinking in a previous fMRI study (Fig 2, [29]). We selected a larger area than that reported by Heatherton et al. in order to account for the lower spatial precision of EEG as compared to fMRI.

Source localisation was performed with linearly constrained minimum variance (LCMV) beamforming [47]. LCMV beamforming is an adaptive spatial filter that attenuates the activity of sources outside the region of interest (ROI), while preserving the activity from the sources within the ROI. The ROI activity *y*[*t*] is estimated as the dot product between the spatial filter w* and the EEG measurements at *N* electrode locations **x**[*t*] ∈ *R*^*N*^: *y*[*t*] = w*^T^ **x**[*t*]. The spatial filter is obtained by solving the optimization problem

$$\begin{array}{c} w^{*}=\arg\;\min_{w}\left\{w^{T}\Sigma_{EEG}w\right\}\;\text{s.t.}\;w^{T}a=1, \end{array}$$ (1)

which has the analytic solution [47]

$$\begin{array}{c} w^{*}={\left(a^{T}\Sigma_{EEG}^{-1}a\right)}^{-1}a^{T}\Sigma_{EEG}^{-1}. \end{array}$$ (2)

Here, Σ_*EEG*_ ∈ *R*^*N*×*N*^ is a spatial covariance matrix of the EEG data computed for every subject from the experiment data (pre-filtered for 1–100 Hz with 3-rd order Butterworth filter); $a\in R^{N}$ is the average topography, calculated by averaging projections on the scalp of the selected MPFC voxels. The voxels projections are provided by the forward model.

#### Statistical testing

**Fig 3:**
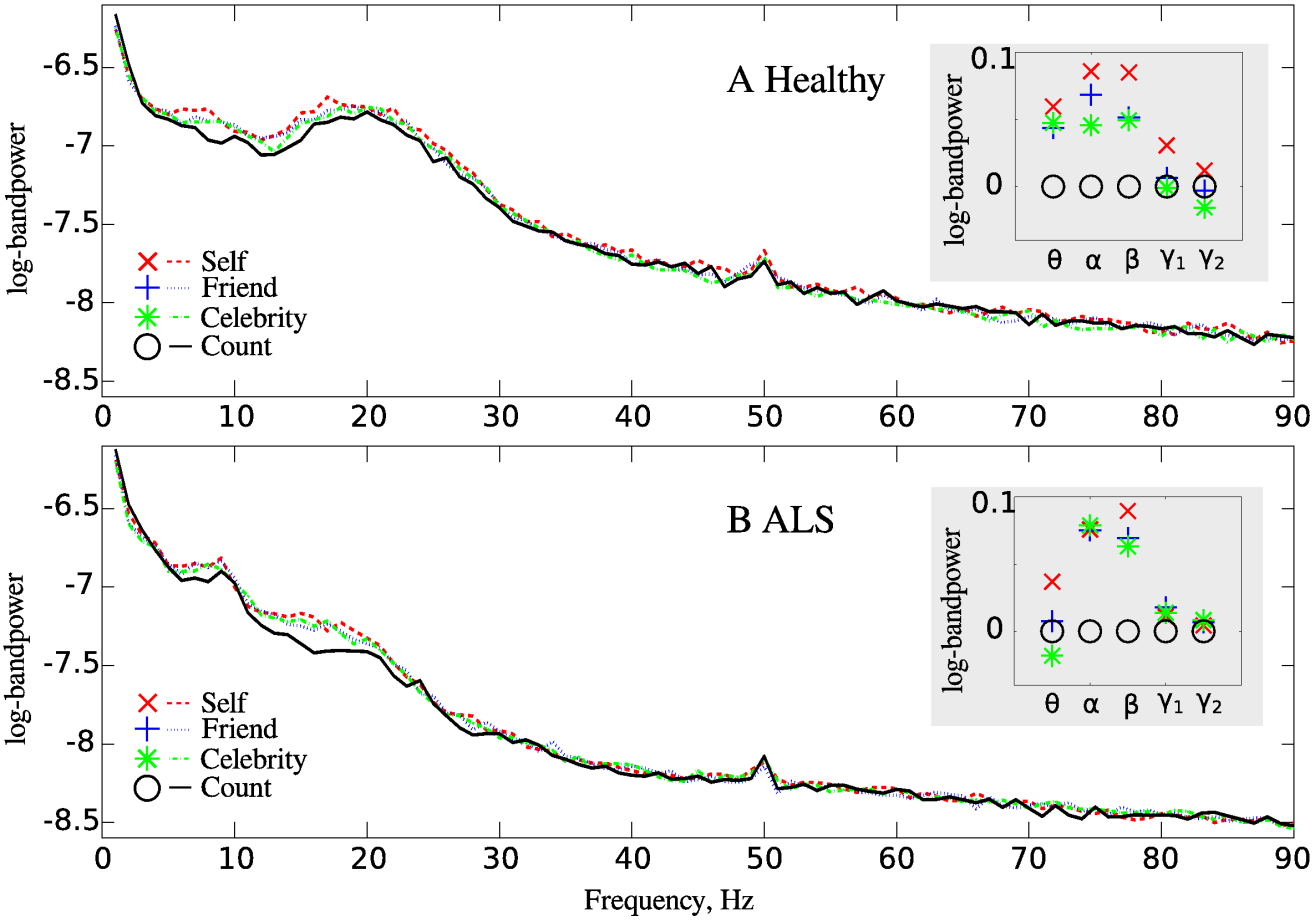
Mean MPFC log-bandpower is modulated by the conditions “self”, “friend”, “celebrity”, “count”. A. Healthy individuals, and B. ALS patients. Inset plot shows for every frequency band the modulation of the mean log-bandpowers in the self-referential conditions relative to the control (“count”) condition. The mean log-bandpower is averaged over subjects and trials for every frequency band. *γ*_1_ indicates low *γ* and *γ*_2_ indicates high *γ*. Note that modulations relative to the control condition were used only for visualisation (and not for the ANOVA).

We computed the log-bandpower estimates of the beamformed signal from the MPFC for every subject and every trial, as now described. Each five-second window after the adjective presentation onset was multiplied with a Hann window, an FFT was performed, and the log-bandpower was calculated and averaged over the individual frequency bands (*θ*, *α*, *β*, low *γ*, high *γ*). For visualisation purposes (Fig 3), we averaged the log-bandpower over the trials and participants for each group (healthy and ALS). We performed the same averaging for log-bandpower in the individual frequency bands and then subtracted the averaged log-bandpower in the control condition from the averaged log-bandpower in every other condition for every frequency band (Fig 3, inset).

We performed analysis of variance (ANOVA) on the log-bandpower averaged over the individual frequency bands using a general linear factorial model. The band power was analysed for healthy individuals and for ALS patients separately in an n-way mixed ANOVA, with condition (“self”, “friend”, “celebrity”) and frequency band (*θ*, *α*, *β*, low *γ*, high *γ*) as within-subjects variables and subject ID as a between-subjects variable. All tests of significance were performed at *α* = .05. For control, we applied an n-way mixed ANOVA to the four conditions (“self”, “friend”, “celebrity”, “count”) in the same way. This allowed us to identify the effects that were specific to self-referential processing and to exclude possible confounding due to the inability of participants to follow the instructions due to general cognitive decline, lack of concentration, misunderstanding of instructions, falling asleep during the experiment, etc. For all ANOVAs, we calculated partial eta squared values ($\eta_{p}^{2}$) as a measure of effect size.

After testing our hypothesis, we performed an additional post-hoc exploratory analysis. Specifically, we pointed a beamformer at each of the 15028 dipoles, computed the log-bandpower estimates for every subject and trial (pre-multiplied with the five-second width Hann window) and performed ANOVA, as described above.

## Results

First, we analysed the differences between the self-referential and control conditions in order to ensure that the participants were able to follow the instructions. Both healthy individuals and ALS patients showed modulations of MPFC log-bandpower for different conditions, with less log-bandpower in the control non-self-referential condition than in the self-referential conditions (Fig 3). We performed an ANOVA to test whether the log-bandpowers averaged over the individual frequency bands (Fig 3, inset) were significantly different for the self-referential (“self”, “friend”, “celebrity”) and control (“count”) conditions. A significant main effect of the condition (“self”, “friend”, “celebrity”, “count”) on bandpower was found both for healthy individuals (*F*(3, 3983) = 9.17, *p* = 0.0000, $\eta_{p}^{2}=0.0137$) and for ALS patients (*F*(3, 3999) = 5.94, *p* = 0.0005, $\eta_{p}^{2}=0.0081$). This agrees with previous EEG, fMRI and PET studies with healthy individuals [28–33], which also found that MPFC activity differs between self-referential and non-self-referential processing. This also suggests that both healthy individuals and ALS patients engage the MPFC differently for self-referential and non-self-referential (control) tasks. Thus, both groups were able to understand and follow the experimental tasks.

To investigate the effect of the degree of self-referentiality on the MPFC EEG, we omitted the control (“count”) condition and performed the ANOVA again. We found that a main effect of the self-referential conditions (“self”, “friend”, “celebrity”) on bandpower remained significant for healthy individuals *F*(2, 2984) = 4.03, *p* = 0.0179, $\eta_{p}^{2}=0.0054$. This agrees with previous EEG, fMRI and PET studies with healthy individuals [28–33], which also found significant modulation of the MPFC by different degrees of self-referential processing. However, a main effect of the self-referential conditions (“self”, “friend”, “celebrity”) on bandpower was not significant for ALS patients: *F*(2, 2984) = 1.26, *p* = 0.2837, $\eta_{p}^{2}=0.0011$. These results are illustrated in Fig 3, inset: In healthy subjects, the ordering of conditions with respect to the depth of self-referential processing is preserved across all frequency bands, i.e., the condition “self” elicits the highest bandpower relative to the baseline condition “count”, followed by the conditions “friend” and “celebrity”. In ALS patients, this ordering is not preserved in the *α* and in the *γ* bands. These results suggest that there are differences in the MPFC activations between healthy individuals and ALS participants in self-referential processing.

**Fig 4:**
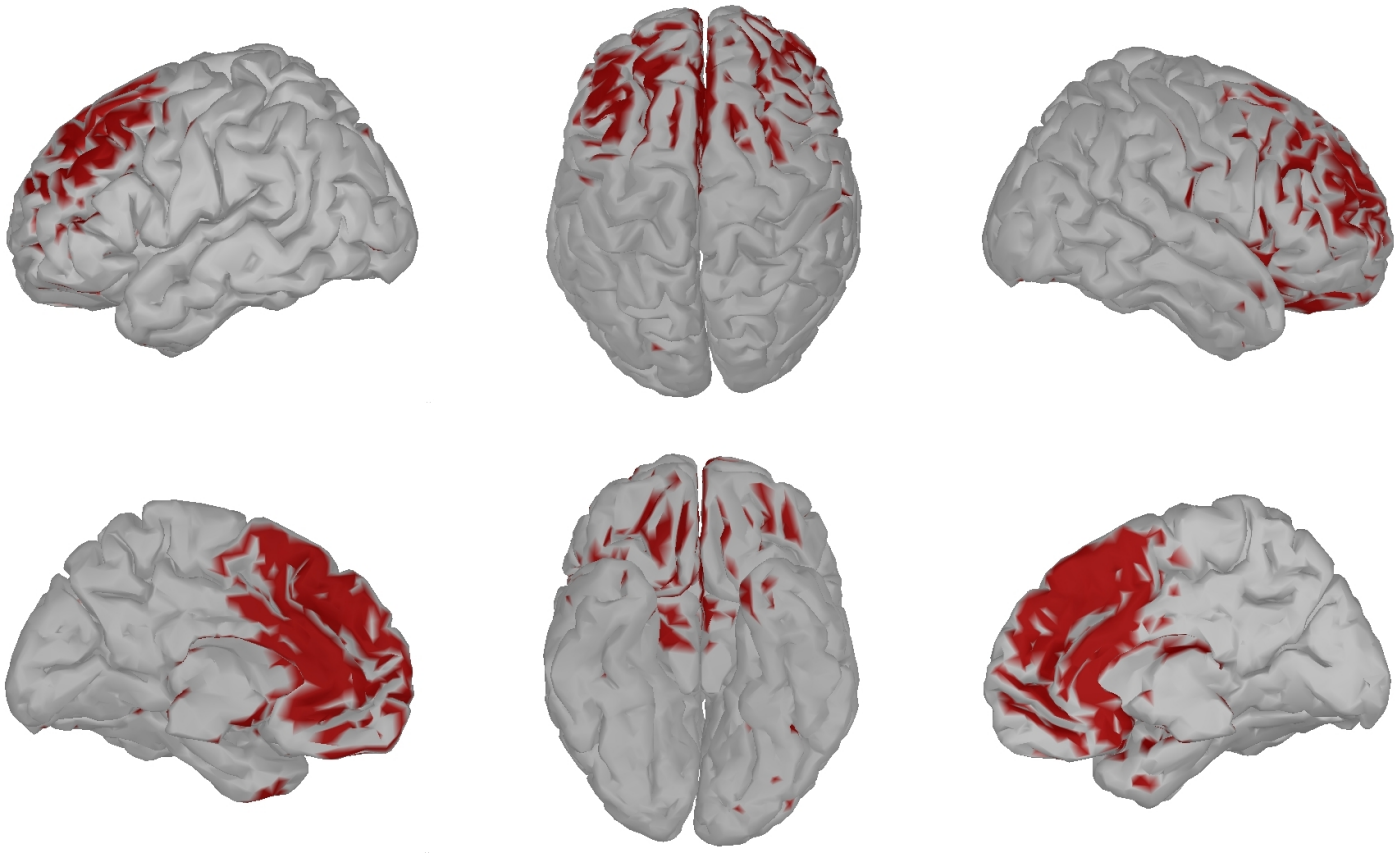
Healthy controls: p-value brain map. Color shows voxels with *p* < 0.05.

**Fig 5:**
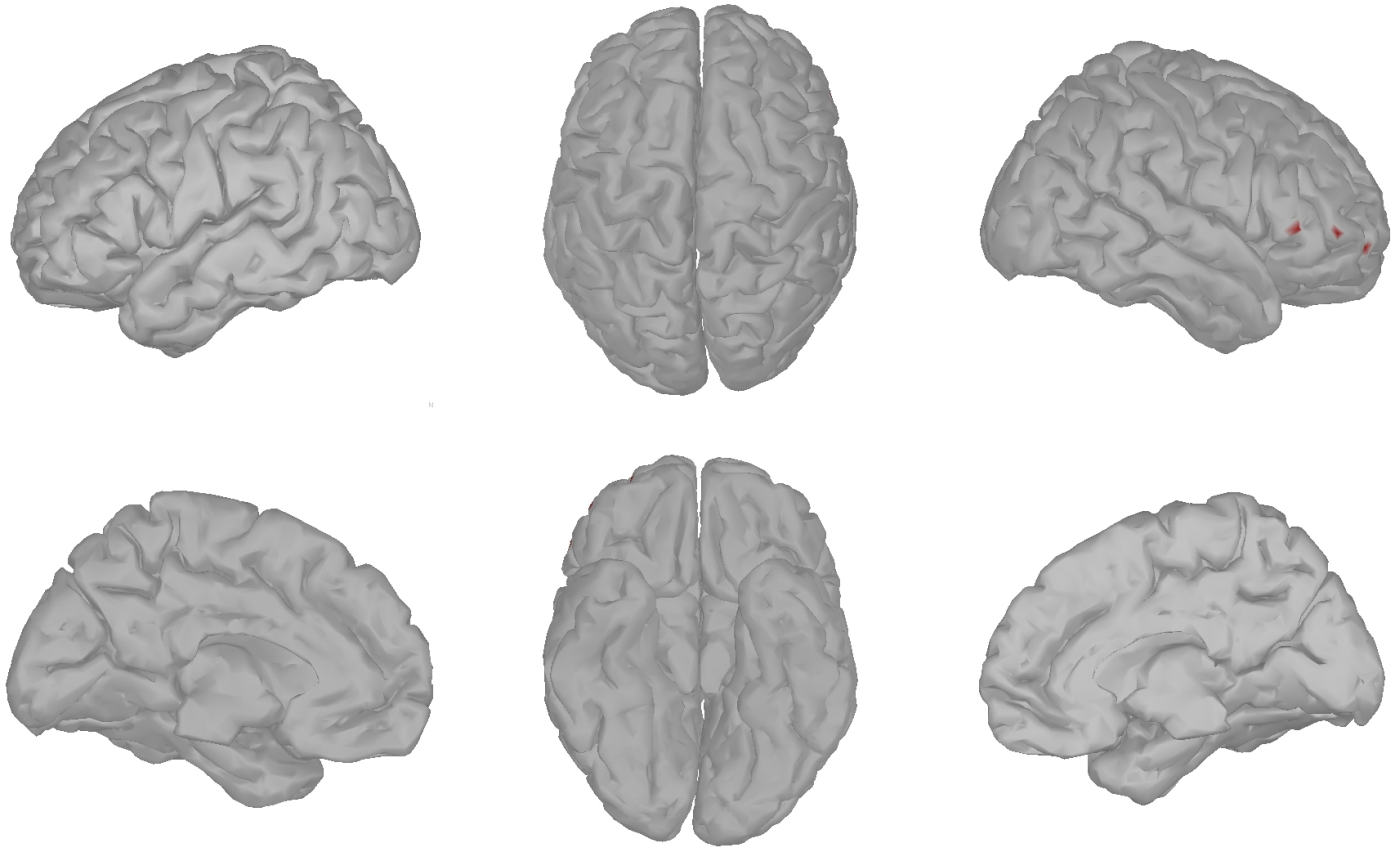
ALS patients: p-value brain map. Color shows voxels with *p* < 0.05.

After testing our hypothesis, we further investigated whether any cortex regions beyond the MPFC are involved in self-referential processing in ALS patients. For this investigation, we performed an ANOVA on log-bandpowers in three self-referential conditions (“self”, “friend”, “celebrity”) for all brain voxels. Figs 4 and 5 show brain areas of healthy individuals and ALS patients for which the main effect of the self-referential condition on log-bandpower has a p-value below 5% (without correction for multiple comparisons). If there is no effect of the depth of self-referential processing on the EEG bandpower, one can expect 5% of the tested voxels to show false-positive results. We observed 10.68% of the voxels to be statistically significant for healthy individuals and 0.02% for ALS patients. For the healthy individuals, the p-values fall below 5% in the MPFC, with the DMPFC being more prominent. The latter agrees with a previous fMRI study that found the DMPFC activity to correlate specifically with trait judgments [31]. This observation lends further support to our hypothesis that there is an effect of depth of self-referential processing on EEG of healthy individuals, but not on EEG of ALS patients.

## Discussion

Our study broadens the scope of cognitive abnormalities in ALS patients. Impaired memory functions and emotion processing have been observed previously in non-demented ALS patients [5, 48, 49]. In combination, these cognitive deficits can give rise to impaired self-awareness (anosognosia) [35]. We tested the hypothesis of altered self-referential processing in ALS, and indeed we found absence of the EEG correlates of self-referential processing in ALS patients.

The observed results cannot be explained simply by impaired attention [49] or the inability of patients to understand a given task. Both patients and healthy individuals showed significantly distinct levels of activation in the MPFC for self-referential and counting conditions. This suggests that both groups understood and were able to perform the task. Thus, the alterations in neural processing that were detected by EEG were specific to self-referential processing and might indicate that ALS patients have difficulties in distinguishing themselves from others.

Our results agree with the serotonergic model of ALS progression [24] discussed in the Introduction (Fig 1). However, we cannot exclude the possibility that the alterations to self-referential processing were caused not by ALS directly, but rather by other confounding factors, which we discuss in the following.

First, long-term paralysis and limitations to act and communicate can change the way one sees oneself and others. Birbaumer et al. suggested that the inability to act leads to thought-extinction [50]. Such thought extinction might be specific to thoughts about oneself, as one cannot observe one’s own actions any more, but one can still observe the actions of others. This hypothesis was proposed by Heilman et al., who argued that the lack of sensory feedback and the inability to observe one’s own body acting can cause anosognosia [51].

Second, depression might also contribute to alterations in self-referential thinking [52]. Although none of the participants in this study had been diagnosed with depression, it should be noted that depression diagnosis with CLIS patients is not possible and thus depression cannot be excluded for all patients who participated in this study. Nevertheless, previous studies found no correlation between depression and cognitive impairment in ALS [8]; another study showed that depression is relatively rare in ALS patients [53].

Third, our results may have been confounded by the preprocessing steps. In particular, we observed fewer cortical ICs for ALS patients than for healthy individuals. As such, it would have been possible to have rejected ICs connected with self-referential thinking for ALS patients. Nevertheless, this would mean that the activity of brain sources correlated with self-referential thinking can be identified as cortical in healthy individuals, but not in ALS patients; this would indicate that EEG correlates of self-referential thinking are altered in ALS.

Finally, our conclusions are based on the interpretation of negative results. It is possible that the EEG correlates of self-referential thinking are not absent, but weakened to the level that they were not detected with our setup. Further studies may address this problem by trying to falisfy our conclusion in a larger patient group.

Altered self-referential processing and lack of self-awareness is associated with a number of neurodegenerative and psychiatric disorders [35]: schizophrenia [36, 54], Alzheimer disease [55, 56], frontotemporal dementia (FTD) [35]. In fact, anosognosia is so common in FTD, that it is used as a major criterion for diagnosis of FTD [57]. Physiologically, FTD is characterised by degeneration of frontal areas that leads to cognitive processing disruption. FTD often co-occurs with ALS and shares genetic correlates with ALS [2, 58]. Although it is usually considered to be an independent disease, there might be a continuum between ALS and FTD, with symptoms of each individual disease being more or less pronounced in different patients [2, 5, 9, 59]. Our results are consistent with the theory of an ALS-FTD continuum since we found alterations in EEG that were related to self-referential processing in ALS patients.

A longitudinal study of 52 patients with sporadic ALS over an 18-month period showed that cognitive deficits progress more slowly than motor deficits [48]. In this case, cognitive deficits, and especially deficits in self-referential thinking, might develop in many ALS patients in the late stages of the disease, in particular after entering the CLIS stage when, due to the lack of communication means, they cannot be detected any more with conventional behavioural tests and questionnaires. With self-referential processing being a key component of consciousness [60], the question arises whether the consciousness of the CLIS ALS patients is also altered. Alterations to consciousness [61] might explain the decreased activation of the DMN observed in ALS patients [27] and the difficulty of communication attempts using Brain-Computer Interfaces (BCI) in CLIS ALS patients [62].

Future studies should address the problem of consciousness in CLIS ALS patients. Even though established fMRI methods for consciousness estimation exist [61], it can be difficult to use these methods with CLIS patients for safety reasons. EEG methods for consciousness estimation, for example entropy estimation, should be used to determine the level of consciousness of CLIS ALS patients [63]. The issue of consciousness in CLIS ALS has implications not only for ALS research and for the developers of BCI systems, but more importantly for patients and their families. This knowledge can affect the patient’s perspective on their disease and influence their end-of-life decisions.
